# Physical activity, sedentary time, and fitness in relation to brain shapes in children with overweight/obesity: Links to intelligence

**DOI:** 10.1111/sms.14263

**Published:** 2022-11-22

**Authors:** Cristina Cadenas-Sanchez, Jairo H. Migueles, Juan Verdejo-Román, Kirk I. Erickson, Irene Esteban-Cornejo, Andrés Catena, Francisco B. Ortega

**Affiliations:** 1 PROFITH “PROmoting FITness and Health through physical activity” Research Group, Sport and Health University Research Institute (iMUDS), Department of Physical and Sports Education, Faculty of Sport Sciences, University of Granada, Granada, Spain; 2 Institute for Sustainability & Food Chain Innovation (IS-FOOD), Department of Health Sciences, Public University of Navarra, IdiSNA, Navarra Institute for Health Research, Pamplona, Spain; 3 Centro de Investigación Biomédica en Red Fisiopatología de la Obesidad y Nutrición (CIBERobn), Instituto de Salud Carlos III, Madrid, Spain; 4 Department of Biosciences and Nutrition at NOVUM, Karolinska Institutet, Huddinge, Sweden; 5 Department of Personality, Assessment & Psychological Treatment, University of Granada, Granada, Spain; 6 Mind, Brain and Behavior Research Center (CIMCYC), University of Granada, Granada, Spain; 7 Brain Aging & Cognitive Health Lab, Department of Psychology, University of Pittsburgh, Pennsylvania, Pittsburgh, USA; 8 AdventHealth Research Institute, Florida, USA; 9 School of Psychology, University of Granada, Granada, Spain; 10 Faculty of Sport and Health Sciences, University of Jyväskylä, Jyväskylä, Finland

**Keywords:** brain shapes, cardiorespiratory fitness, cognitive performance, gray matter, moderate-to-vigorous, muscular strength, sedentarism, speed-agility

## Abstract

**Objectives:**

To examine the association between physical activity, sedentary time, and physical fitness with the shapes of subcortical brain structures in children with overweight/obesity. Further, we analyzed whether differences in the shapes of subcortical brain structures were related to intelligence. We hypothesized that those children with higher physical activity levels, lower sedentary time, and better fitness, would show greater expansion of the brain regions analyzed, and these expansions would be associated with higher intelligence.

**Study Design:**

100 children (10.0 ± 1.1 years, 40 girls) were included in the analyses. Physical activity and sedentary time were measured by accelerometry, and physical fitness was evaluated by a fitness battery. Shapes of subcortical brain structures were assessed by magnetic resonance imaging. Intelligence was measured by the Kaufmann Brief Intelligence test.

**Results:**

Physical activity was related to expansion of the right/left pallidum, right/left putamen, and right thalamus (*p* < 0.05). Higher sedentary time was related to contraction of the left thalamus and right nucleus accumbens (*p* < 0.05). Higher levels of cardiorespiratory fitness were associated with expansion of the right amygdala (*p* = 0.022). Greater strength in the upper-limb was related to expansion of the right/left pallidum and the left nucleus accumbens (*p* < 0.038), and contraction of the left amygdala (*p* = 0.030). Better speed-agility was associated with expansion of the left nucleus accumbens (*p* = 0.036). Physical activity- and fitness-related expansion of the right pallidum was associated with higher intelligence (all *p* < 0.05).

**Conclusion:**

Physical activity, sedentary time, and physical fitness were significantly related to the shapes of subcortical brain structures, which in turn were related to intelligence in children with overweight/obesity.

## 1 | INTRODUCTION

Childhood represents a period of rapid growth and brain development characterized by heightened neuronal plasticity.^1^ However, greater childhood adiposity could affect brain development since obesity is related to differences in brain morphology and poorer cognitive performance compared to normal-weight individuals.^2^ Indeed, maintaining or improving brain health is important for developmental outcomes including cognitive performance and academic achievement.^3^ In contrast to the negative associations between adiposity and brain health, there is evidence that greater amounts of physical activity and higher physical fitness levels might be beneficial for brain health.^3,4^

Physical activity is related to lower risk of mortality and beneficial cardiovascular health outcomes.^3,5^ Yet, there are inconsistent associations between physical activity and brain health, especially at certain points of the lifespan.^6,7^ Further, there is limited information available about the association between sedentarism and brain outcomes.^3^ We previously observed that sedentary time and sedentary behaviors were related to lower gray matter volume.^8,9^ In contrast to sedentary behaviors, physical fitness (i.e., cardiorespiratory fitness, muscular strength, and speed-agility), a physiological construct that is closely linked to physical activity behaviors, is considered an important marker of health throughout the lifespan.^10,11^ A mounting body of evidence has shown that cardiorespiratory fitness is positively associated with greater brain volumes across the lifespan.^12,13^ For instance, we found that cardiorespiratory fitness and speed-agility, but not muscular strength, were associated with higher regional gray matter volume in children with overweight/obesity.^12,13^

While previous studies have focused on volumetric analyses, only one study^14^ in children has examined whether fitness is associated with subcortical local expansions and contractions of certain brain areas (i.e., shape analysis). Indeed, using this approach, which allows the detection of locally precise changes in brain morphology of various brain regions, all physical fitness components (i.e., cardiorespiratory fitness, muscular strength, and speed-agility) were significantly associated with the shape of several subcortical structures in normal-weight children.^14^ In particular, higher levels of physical fitness were associated with expansions (i.e., pallidum, putamen, thalamus, nucleus accumbens, amygdala, caudate nucleus, and hippocampus) but also contractions (i.e., pallidum, putamen, thalamus, nucleus accumbens, amygdala, and hippocampus) of subcortical brain structures.^14^ To the best of our knowledge, there is no evidence examining the relationship between physical activity, sedentary time, and the shape of subcortical brain structures in children. Such findings could have significant public health and educational significance because the shape of subcortical structures is considered as an index of neural development^15^ and is related to measures of cognition.^16^ As such, determining whether behavioral (physical activity and sedentary time) and physiological (physical fitness) factors are related to indices of neural development and intelligence could help guide the development of early intervention strategies for enhancing brain and cognitive outcomes.

Therefore, the present study aimed to examine the relationship between physical activity, sedentary time, and physical fitness components with the shapes of subcortical brain structures in children with overweight/obesity. Further, we examined whether any subcortical expansions and contractions was related to intelligence. We hypothesized that those children with higher physical activity levels, lower sedentary time, and better fitness would show greater expansion of the brain regions analyzed, and these expansions would be associated with higher intelligence.^17^

## 2 | METHODS

### 2.1 | Study design and study participants

This study reports secondary analyses using the baseline data collected from the ActiveBrains project (http://profith.ugr.es/activebrains, registration no. NCT02295072), a randomized controlled trial that aims to investigate the effect of a 20-week exercise intervention on brain health and physical health outcomes in children with overweight/obesity.^18^ The detailed protocol (e.g., study randomization, recruitment procedure, inclusion and exclusion criteria, rationale of the sample selected, and measurements) of the study has been previously described.^18^

Initially, 110 children registered in the study. Those with valid physical activity, sedentary time or physical fitness and brain data were included in this cross-sectional analysis (n = 100, 10.0 ± 1.1 years, 40 girls). Parents or legal guardians were informed of the aims of the project and signed written informed consent. This study was approved by the Ethics Committee on Human Research of the University of Granada.

### 2.2 | Measurements

#### 2.2.1 | Physical activity and sedentary time

Accelerometer data collection and data processing are described elsewhere.^19^ Briefly, participants were asked to wear ActiGraph GT3X+ accelerometers (ActiGraph, Pensacola, FL, USA) on their non-dominant wrist for 7 consecutive days (24 h). A sleep log with information on time to bed and time out of bed was fulfilled. Accelerometers were initialized to record accelerations at 100 Hz. Raw accelerations were downloaded via the ActiLife software and processed in the R packaged GGIR (v.1.5.12).^20^ Non-wear time and non-standard high accelerations related to malfunctioning of the accelerometers were imputed by average acceleration during the same time interval from the rest of the days.^20^ Finally, sedentary time (<35 mg), light physical activity (LPA: 35–199 mg), moderate physical activity (MPA: 200–699 mg), vigorous physical activity (VPA: >700 mg), and moderate-to-vigorous physical activity (MVPA: ≥200 mg) were obtained.^21,22^ The participants were excluded if they recorded less than 4 valid days (≥16 h/day), including at least 1 weekend day.

#### 2.2.2 | Physical fitness

Cardiorespiratory fitness, muscular strength, and speed-agility were measured by the ALPHA (Assessing Levels of Physical Activity) fitness test batteiy which is feasible, reliable, and valid for children and adolescents.^23^ In brief, cardiorespiratory fitness was measured by a 20 m shuttle run test. This test was performed only once and in the last position. Maximum oxygen consumption (VO_2_max) was calculated using the Leger et al.^24^ equation and used for analyses. Upper-limbs muscular strength was measured by the handgrip strength test, and lower-limbs muscular strength was evaluated by standing long jump test. Speed-agility was assessed by the 4×10 m shuttle run test. For the analyses, we inverted the speed-agility variable by multiplying the test completion time in seconds by −1. Thus, higher scores indicate better performance.

#### 2.2.3 | Intelligence

The Kaufman Brief Intelligence Test (K-BIT) was used for assessing intelligence. The K-BIT consisted of two subscales, vocabulary and matrices, which measured crystallized and fluid intelligence, respectively. The vocabulary scale assesses language and the ability to use experience-related knowledge. The matrices scale evaluates abstract reasoning skills and problem solving. The K-BIT was individually administered by experienced evaluators. Age-specific norms were used to calculate crystallized, fluid and total intelligence, which were used for the analyses.

#### 2.2.4 | Magnetic resonance imaging: Data acquisition and data processing

High resolution structural brain images were acquired from magnetic resonance imaging (Siemens Magnetom Tim Trio, 3 T, Siemens, Germany). We collected a high resolution sagittal three-dimensional T1-weighted image using a magnetization-prepared rapid gradient-echo sequence (MPRAGE). The total duration of the T1 sequence was 6 min and 34 s for each child.

FSL/FIRST (http://fsl.fmrib.ox.ac.uk/fsl/fslwiki/FIRST) software was used for segmentation of subcortical structures and shape analysis. FIRST is a tool that segment a set of fifteen subcortical brain structures: brain stem and right and left pallidum, putamen, thalamus, nucleus accumbens, amygdala, caudate nucleus, and hippocampus.

Shape analysis is based on the individual meshes composed by a large number of vertices and triangles. The number of vertices and triangles is the same for each structure, which in turns allow intra- and inter-participant comparison of each vertex. These comparisons are possible because all meshes are aligned to the Montreal Neurological Institute space and pose (rotation and translation) is removed. More information related to the vertex-wise methods is described elsewhere.^16^ To assess local changes in each region, we used the radial distance of each vertex to the medial curve of the structure (the centroid curve of the boundary in each section). Regional expansions and contractions (i.e., radial distances related to each vertex spatial location to the core line of the structure) of the region are the indicators of local changes in the structure shape.

#### 2.2.5 | Covariates

Maturational status was measured by peak height velocity. It was derived from standing and/or seated height using the Moore et al.^25^ equations. Highest parental education was self-r eported and therefore categorized as both of them, one of them, or neither of them reaching university-level education.

#### 2.2.6 | Statistical analyses

A partial correlation permutation approach was used to examine the association between physical activity (i.e., LPA, MPA, VPA, and MVPA), sedentary time, physical fitness components (i.e., cardiorespiratory fitness, upper-limb muscular strength and lower-limb muscular strength, and speed-agility), and the shapes of brain subcortical structures. Therefore, the correlation between the radial distance of each vertex and each independent variable (i.e., physical activity, sedentary time, and physical fitness) was computed after adjusting for sex, estimated timing of peak height velocity, and parental education. We also tested whether the associations found were independent of body mass index. Additionally, we explored whether our findings on sedentary time were independent of MVPA.

Then, we examined whether the subcortical brain structures associated with physical activity, sedentary time, or physical fitness, were also related to total, crystallized or fluid intelligence. For such purpose, we first applied the partial correlation permutation approach to examine the association of intelligence with the shapes of brain structures adjusting for sex, estimated timing of peak height velocity, and parental education; then, we created specific output masks with those voxels that were significantly correlated with physical activity, physical fitness, and intelligence; and lastly, we overlapped the outputs for examining the expansion or contractions of the brain shapes that were concomitantly associated with either physical activity or physical fitness and intelligence.

To control for multiple comparisons, we used the threshold-free cluster enhancement approach. Results are presented as color-coded significance maps. We used blue colors to indicate a negative outcome-predictor association (i.e., the higher the value of the predictor, the smaller are radial distances), orange colors to indicate positive associations (i.e., the higher the value of the predictor, the larger are radial distances), and gray color to show non-significant associations. *p* values for all neuroimaging analyses were established as *p* < 0.05 corrected using the threshold-free cluster enhancement approach.

## 3 | RESULTS

### 3.1 | Physical activity and sedentary time

Characteristics of the study participants are shown in Table S1. The accelerometer data compliance showed that 90.5% of the participants wore the accelerometer device for 7 consecutive days, and out of the 9.5% remaining (n = 2) had less than 4 valid days, and therefore, were excluded for not meeting the minimum recommended.^26^

**FIGURE 1:**
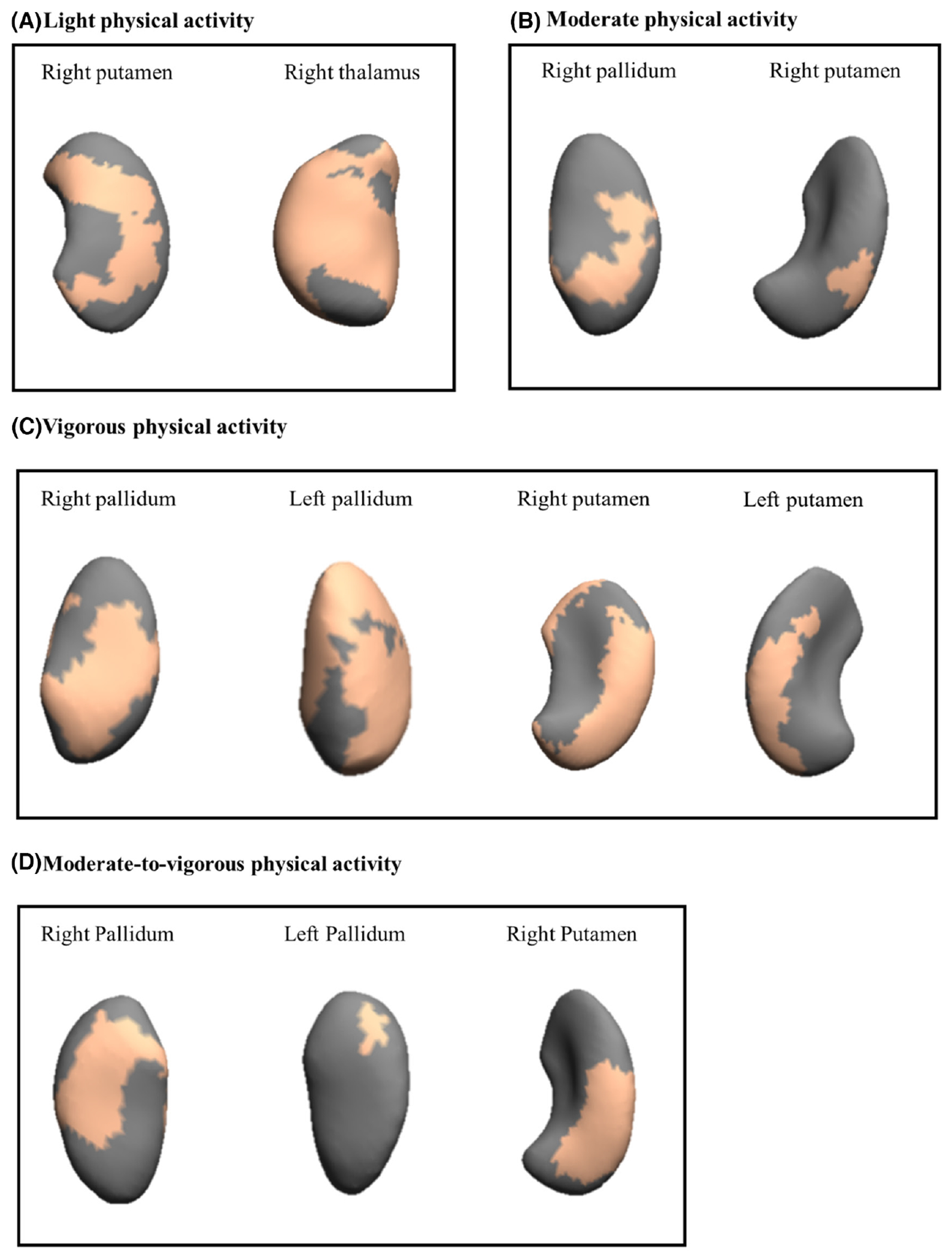
Mappings of significant expansions of subcortical regions related to physical activity. The orange color indicates the significance threshold-free cluster enhancement corrected *p* values (*p* < 0.05). Gray color indicates no association. All the analyses were adjusted for sex, estimated timing of peak height velocity, and parental education.

**FIGURE 2:**
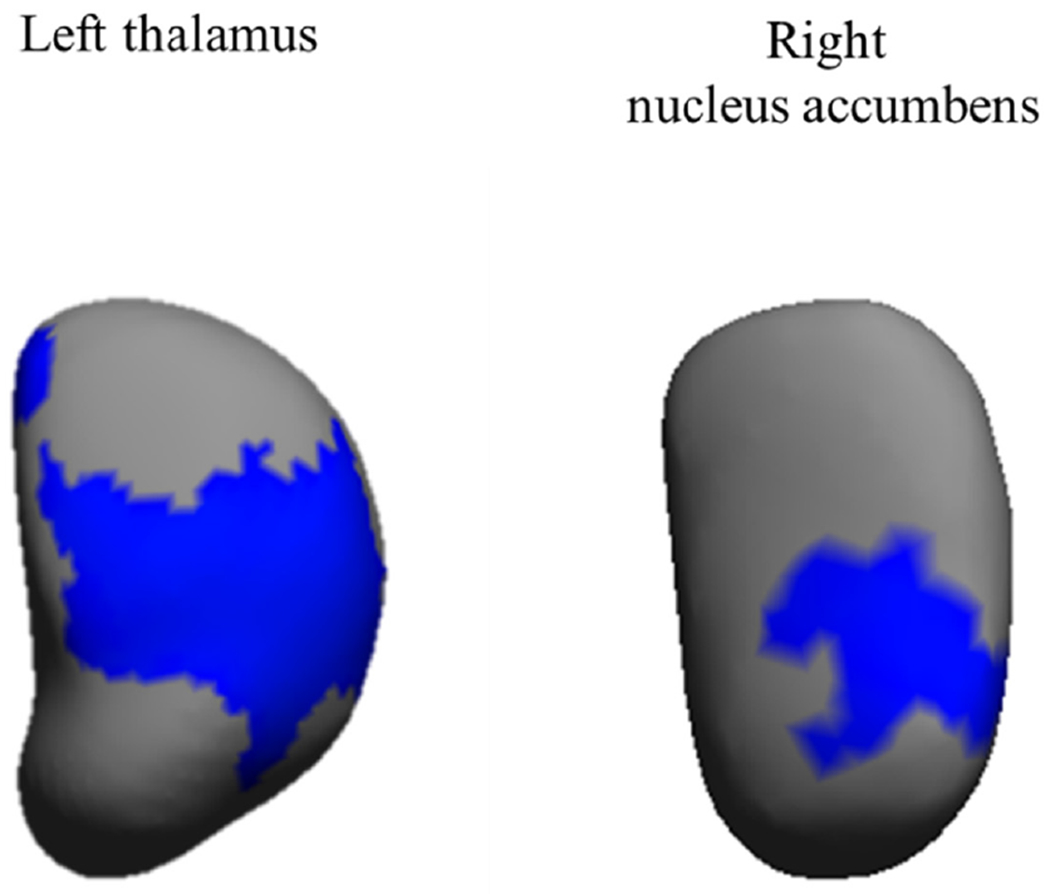
Mappings of significant contractions of subcortical regions related to sedentary time. The blue color indicates the significance threshold-free cluster enhancement corrected *p* values (*p* < 0.05). Gray color indicates no association. All the analyses were adjusted for sex, estimated timing of peak height velocity, and parental education.

Results from the vertex-wise permutation tests for physical activity and sedentary time with subcortical structural shapes (radial distances) adjusted for sex, estimated timing of peak height velocity, and parental education can be found in Table S1. We observed that measures of physical activity (i.e., LPA, MPA, VPA, and MVPA) were significantly related to the shape expansions of the right/left pallidum, right/left putamen, and right thalamus (17 to 2679 voxels, all *p* < 0.05). Of the physical activity intensities studied, VPA showed the greatest number of positive (i.e., expansions) significant associations with subcortical structural shapes (four out of fifteen regions studied). No significant association was observed for nucleus accumbens, amygdala, caudate nucleus, and hippocampus (all *p* > 0.05). Same results were observed when the associations were further adjusted for body mass index (data not shown). Sedentary time was significantly related to the contraction of left thalamus and right nucleus accumbens (10 to 1273 voxels, all *p* < 0.05, Table 1). We did not observe any significant association to pallidum, putamen, amygdala, caudate nucleus, and hippocampus. After additional adjustment for body mass index, the significant findings observed disappeared (all *p* > 0.05). When we explored whether sedentary time was independent of MVPA, the significant results remained similar (borderline non-significant for left thalamus, and significant association for right nucleus accumbens; all *p* < 0.08). Figure 1 and Figure 2 graphically show how physical activity and sedentary time variables relate to the shape of each region studied, by means of a color-coded significance map, the part of each region that was significantly related to physical activity and sedentary time, and the direction of the association (orange color: expansions, blue color: contractions, and gray color: no association).

### 3.2 | Physical fitness

**FIGURE 3:**
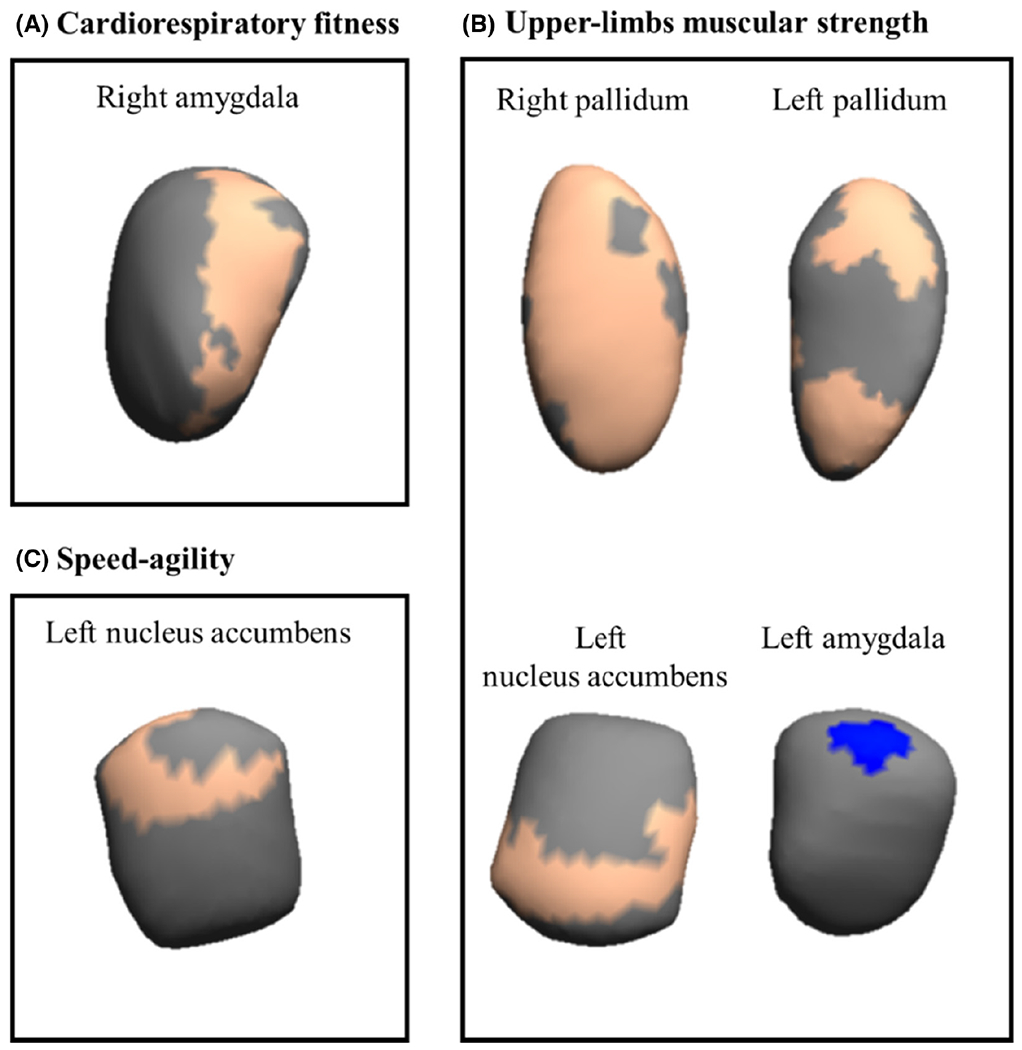
Mappings of significant (expansions/contractions) subcortical regions related to physical fitness. The color indicates the significance threshold-free cluster enhancement corrected *p* values (*p* < 0.05), with blue indicating significant negative associations (i.e., contractions), and orange depicting significant positive association (i.e., expansions) between physical fitness components and brain regions. Gray color indicates no association. All the analyses were adjusted for sex, estimated timing of peak height velocity, and parental education.

Vertex-wise permutation values for each physical fitness component with subcortical structural shapes adjusted for confounders (i.e., sex, estimated timing of peak height velocity, and parental education) can be found in Table 1. Cardiorespiratory fitness was positively associated with right amygdala, that is, those children with higher levels of VO_2_max showed greater expansions of the right amygdala (175 voxels, *p* = 0.022). Upper-limb muscular strength was mainly related to expansions of three regions (i.e., right/left pallidum, and left nucleus accumbens; 124 to 867 voxels, all *p* < 0.038), and contraction in one region (i.e., left amygdala, 48 voxels, *p* = 0.030). Lower-limb muscular strength was not significantly related to the shape of any region (all *p* > 0.05). Speed-agility was associated with expansion of only one region (i.e., left nucleus accumbens; 86 voxels, *p* = 0.036). No significant difference was observed for the remaining brain structures analyzed (i.e., putamen, thalamus, caudate nucleus, and hippocampus; all *p* > 0.05). Figure 3 graphically shows how physical fitness variables relate to shape of the region studied, and the direction of the association (orange color: expansions, blue color: contractions, and gray color: no association). We additionally explored whether physical fitness associations with brain shapes could be independent of body mass index. After additionally adjusting for body mass index, the results remained similar except for speed-agility in relation to the expansion of the left nucleus accumbens (*p* = 0.06) and for upper-limb muscular strength in relation to the expansion of the left pallidum (*p* = 0.08) where the associations were borderline non-significant.

### 3.3 | Intelligence

**FIGURE 4:**
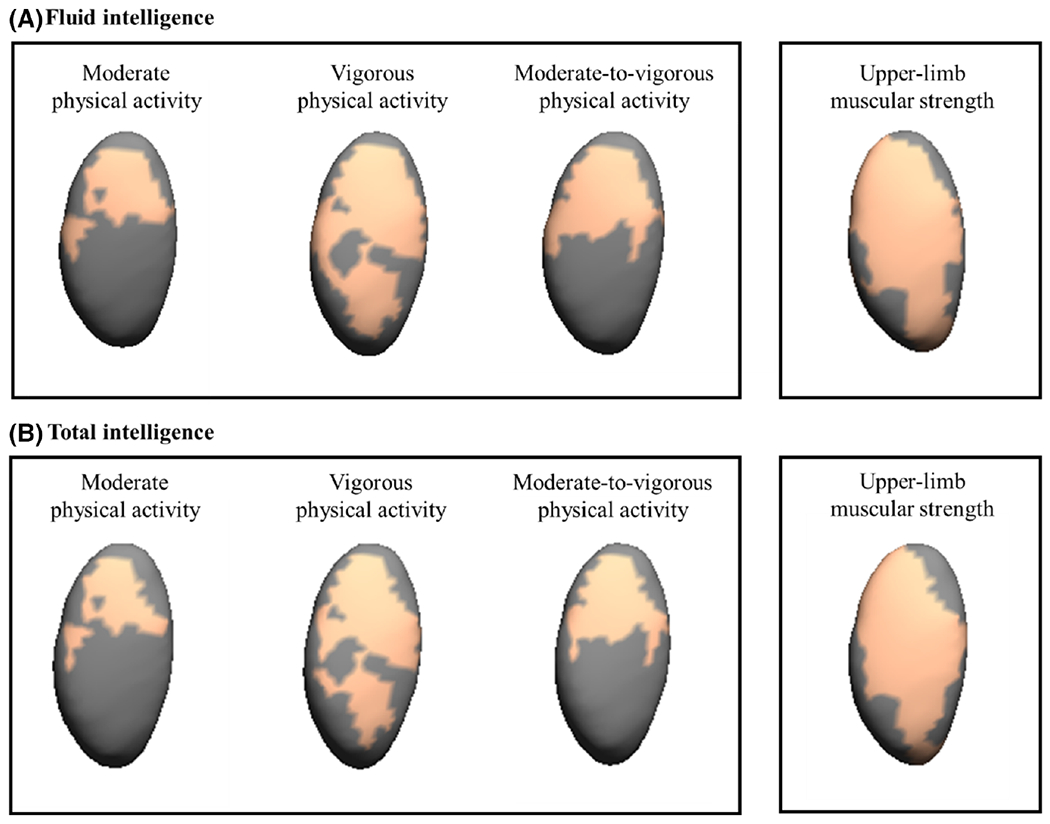
Mappings of expansions in the right pallidum which concomitantly associate with physical activity or upper-limb muscular strength and intelligence. The orange color indicates the significance threshold-free cluster enhancement corrected *p* values (*p* < 0.05). Gray color indicates no association. All the analyses were adjusted for sex, estimated timing of peak height velocity, and parental education.

Figure 4 and Table S2 present the expansions of right pallidum related to intelligence adjusted for sex, estimated timing of peak height velocity, and parental education within those regions previously significant related to physical activity, sedentary time, and physical fitness. We observed expansions in voxels in the right pallidum that were related to upper-muscular strength, moderate and vigorous physical activity and moderate-to-vigorous physical activity to be significantly associated with fluid and total intelligence (2–615 voxels for fluid intelligence and 2–532 voxels for total intelligence).

## 4 | DISCUSSION

Our main finding suggests that physical activity (i.e., LPA, MPA, VPA, and MVPA) and physical fitness components (i.e., cardiorespiratory fitness, upper-limb muscular strength, and speed-agility) were mainly related to expansion of several subcortical brain structures (i.e., pallidum, putamen, thalamus, nucleus accumbens, and amygdala), while sedentary time was associated with contractions of subcortical brain structures (i.e., thalamus and nucleus accumbens) in children with overweight/obesity. No significant relationships were observed for brain stem, caudate nucleus, and hippocampus. Further, we also observed that of these significant regions, expansion of the right pallidum was associated with greater intelligence. Specifically, it could be hypothesized that performing MPA and VPA, as well as having higher upper-body strength, could be related to the right pallidum, and in turn, this could be related to significant associations with intelligence for children with overweight/obesity. However, caution is advised given the cross-sectional nature of this study, and future prospective observational studies and randomized controlled trials should corroborate this hypothesis.

### 4.1 | Physical activity, sedentary time, and physical fitness

Physical activity has been associated with a range of brain health benefits.^3^ However, the latest systematic reviews and meta-analyses published on this topic in children showed mostly inconsistent findings with brain structure.^6,27^ Increasing physical activity levels in children has been considered as a low-cost effective strategy for altering brain health outcomes and has received great interest worldwide due to the alarming data of insufficient physical activity (i.e., >70% do not meet the physical activity recommendations) in youth.^28^ Our novel study supports the idea of increasing physical activity levels, particularly high intensity activities (i.e., vigorous) seem to be related to brain morphology and intelligence. Indeed, we observed that higher levels of physical activity at any intensity were related to expansion of the pallidum, putamen, and thalamus, independently of body mass index. VPA was associated with expansion of a greater number of regions, with a total of four subcortical brain structure studied. To the best of our knowledge, there was no previous study examining associations between physical activity and shapes of subcortical structures which hampers our ability to compare our findings with those from other studies. Previous studies showed inconclusive findings in adolescents but focused on whole-brain approaches or regional volumetric analyses, and used objective and subjective physical activity measurements.^29,30^ In line with this approach, we recently observed that increasing MVPA was associated with greater gray matter volume in the right hippocampus in children with overweight/obesity.^8^ Yet, no significant association was found for hippocampus in the present study. A recent systematic review and meta-analysis^3,6,7^ that included both randomized controlled trials and cross-sectional studies, concluded that there is moderate evidence that physical activity is beneficial to cognition and weak evidence for its effects on brain structure and function during preadolescence. Indeed, a critical evaluation of systematic reviews on physical activity and brain in youth indicates inconsistent findings and few studies examining physical activity related brain changes.^7^ Therefore, further randomized controlled trials are needed to shed light on the relevance of physical activity for brain outcomes, although our findings show promising associations of physical activity of high intensity with measures of brain health, specifically expansions of several subcortical brain regions.

Sedentary behaviors are highly prevalent and have been considered devastating to health.^31^ However, whether sedentary time is related to brain health in children is largely unknown. Two studies from our cohort have shown a negative association of sedentary behavior with brain structure based on regional analyses, using device-based^8^ or questionnaire-based data.^9^ These results are in line with our findings, where sedentary time was mostly related to contractions in brain shapes. Interestingly, we further observe that sedentary time might be dependent of body mass index, but not on MVPA, already at childhood. Our exploratory analyses suggest that the associations were more robust for MVPA which were linked to brain shapes independently of body mass index, while the associations for sedentary time disappear after adjustment for body mass index.

Physical fitness is considered an important marker of brain health.^11^ Our findings inform the relationship between physical fitness components and shape of subcortical brain structures, independently of body mass index. These associations were mainly positive for cardiorespiratory fitness, upper-limb muscular strength, and speed-agility, indicating that higher physical fitness levels in childhood are associated with expansion in several parts of the pallidum, nucleus accumbens, and amygdala. However, upper-l imb muscular strength was also associated with contractions in the left amygdala. These findings are partially in line with the study^14^ analyzing physical fitness components in relation to the shapes of subcortical structures in youth. Particularly, Ortega et al.^14^ examined a sample of pre-adolescent children with normal weight from the NUHEAL project and concluded that higher levels of physical fitness in childhood were related to both expansions and contractions in the pallidum, putamen, thalamus, nucleus accumbens, amygdala, caudate nucleus, and hippocampus. According to our findings, Ortega et al.^14^ observed that upper-limb muscular strength was the most consistently associated outcome with the subcortical brain structures analyzed (NUHEAL: six vs. ActiveBrains: three subcortical brain structures). Then, cardiorespiratory fitness was the second consistently related to shapes of subcortical structures, followed by speed-agility in both projects. Although the order of relevance of the physical fitness components was the same for both studies, the number of significant physical fitness-related regions differ in favor to those of normal-weight children. For instance, the previous study found physical fitness-related associations in putamen, thalamus, caudate nucleus, and hippocampus while we did not. The weight status (NUHEAL: normal weight, mean body mass index of 19.3 kg/m^2^ vs. ActiveBrains: overweight/obesity, mean body mass index of 26.7 kg/m^2^), the physical fitness level (i.e., NUHEAL presents overall 5.2% higher physical fitness level than ActiveBrains sample), and the confounders used in the analyses could explain the differences of the consistency and magnitude of the associations. Previous literature focused on regional volumetric analyses in children observed that those children categorized as higher fit presented higher pallidum volume as we observed in our results.^32^ They also tested whether higher fit children presented larger nucleus accumbens volume, showing a positive trend (but not significant) in favor of fitter kids.^32^ The information in regard to physical fitness and amygdala is lacking in children. Yet, previous literature in adults indicates that the connectivity of the amygdala could be related to participation in an exercise program,^33^ given its role in the processing of emotion, learning, memory, and autonomic cardiovascular adjustments.^34^

Possible mechanisms such as neurotrophic factors could explain the physical activity and physical fitness associations found in this study. Previous literature has shown that brain-derived neurotrophic factor (BDNF) could play a role in synaptic plasticity and development, neuronal transmission, and angiogenesis.^35^ Besides BDNF, insulin-like growth factor 1 and vascular endothelial-derived growth factor are other molecules that are related to neural growth and survival.^35^ Understanding the role of physical activity on these neurotrophic factors, systematic reviews and meta-analyses in humans have confirmed that physical activity has a positive effect on BDNF levels.^36^ In line with this assumption, a recent study from this cohort showed that LPA, MPA, and MVPA were positively associated with BDNF concentrations, and only LPA to vascular endothelial-derived growth factor.^37^ There is a need for randomized controlled trials designed to test the mediating effect of BDNF in the relationship between physical activity and brain structure in children with overweight/obesity.

### 4.2 | Brain shape and intelligence

Few studies have explored the relationship between cognitive function and brain volume.^4,12,38,39^ To the best of our knowledge, only one study^16^ has addressed the association between expansion/contraction of subcortical brain structures and intelligence in preadolescent children. Our findings demonstrate, for the first time, a relationship between expansions of the right pallidum and intelligence in children with overweight/obesity. These finding are remarkable since intelligence is reliant on the optimal functioning of many brain areas including subcortical structure of the basal ganglia.^40,41^ Accordingly, Sandman et al.^16^ showed that expansion of the head and tail of the putamen was associated with higher intelligence scores. Yet, they also observed that larger basal ganglia and medial dorsal putamen volumes were associated with impaired cognitive function in 50 children (6–10 years). Discrepancies between studies could be due to the different characteristics of the study participants (age range, sample size) and methodology applied. Similarly to our analyses, other studies in children and adolescents (from 5 to 18 years) indicated that the intelligence quotient explained about 10% of the variation in gray matter volume, mainly in the cingulate cortex in adolescents.^38^ In a similar sample, Luders et al. found that higher intelligence scores (total, verbal, and performance) were associated with cortical thickness across a wide network of brain areas in an adolescent sample.^39^ Additionally, in this cohort, we have observed that both thicker cortex and gray matter volume (regions previously related to sedentary behavior) were associated with higher intelligence scores.^4,9^ Understanding how brain structure and various aspects of morphology are related to cognition and intellectual functioning is an important area for future research to continue to pursue.

This study also has several limitations. The cross-sectional design of the study does not permit us to make causal relationships between physical activity, sedentary behavior, physical fitness, intelligence, and the shape of subcortical regions. In addition, the inclusion of children with overweight/obesity limits the generalizability of our results to normal weight children. Moreover, the use of a determined cutoff points seems to have a higher influence in the final estimations, and therefore, we need to be cautious when comparing our data. Nevertheless, we further explored the use of different cut-points and placements that were consistently associated with different outcomes in this study sample.^42^ Strengths of this study include a novel and comprehensive analysis of physical activity, sedentary time, and physical fitness with shape of subcortical brain structures in children with overweight/obesity; the objective nature of the measures used (e.g., accelerometers, objective physical fitness tests, magnetic resonance imaging, and standardized tests); the inclusion of standardized measurement of intelligence to enhance the understanding of the importance of expansions and contractions of the subcortical brain structures; and the large sample size of children with magnetic resonance imaging data.

### 4.3 | Perspective

Our findings indicate that higher levels of physical activity and physical fitness, and lower levels of sedentary time, were related to expansions in the shape of subcortical brain structures (i.e., pallidum, putamen, thalamus, and nucleus accumbens) which is interpreted as greater gray matter development in these regions. Moreover, expansions of the right pallidum were associated with higher intelligence scores in children with overweight/obesity. Our results indicate that VPA was the intensity associated with expansion in more subcortical brain regions compared to lower intensities, suggesting that VPA could be particularly beneficial for brain development at early ages. Future randomized controlled trials are needed to confirm or contrast these findings.

## Supplementary Material

Supp Table 1

## Figures and Tables

**TABLE 1:** Brain regions showing a significant correlation between physical activity, sedentary time, physical fitness, and subcortical brain shape

	Contrast	Voxels	*p*
*Light physical activity*			
Right putamen	Expansions	601	0.036
		337	0.037
Right thalamus	Expansions	2679	0.007

*Moderate physical activity*			
Right pallidum	Expansions	310	0.028
Right putamen	Expansions	99	0.045

*Vigorous physical activity*			
Right pallidum	Expansions	636	0.007
Left pallidum	Expansions	886	0.013
Right putamen	Expansions	1808	0.004
Left putamen	Expansions	598	0.035
	Expansions	17	0.049

*Moderate-to-vigorous physical activity*			
Right pallidum	Expansions	435	0.015
Left pallidum	Expansions	41	0.049
Right putamen	Expansions	566	0.023

*Sedentary time*			
Left thalamus	Contractions	1273	0.030
		10	0.049
Right nucleus accumbens	Contractions	47	0.040

*Cardiorespiratory fitness*			
Right amygdala	Expansions	175	0.022

*Upper-limb muscular strength*			
Right pallidum	Expansions	867	0.008
Left pallidum	Expansions	353	0.038
		124	0.037
Left nucleus accumbens	Expansions	195	0.017
Left amygdala	Contractions	48	0.030

*Speed-agility* ^a^			
Left nucleus accumbens	Expansions	86	0.036

*Note*: Expansions (i.e., positive associations) indicate larger radial distance, and contractions (i.e., negative associations) show shorter radial distance in the regions studied (*p* < 0.05 threshold-free cluster enhancement corrected). No significant correlations between other sedentary time, physical activity, physical fitness variables, and the remaining brain shape regions studied are not included in this table. All the analyses were adjusted for sex, peak height velocity, and parental education.

a For analyses, the time spent in the speed-agility test (less time indicating a faster runner) was multiplied by −1, so that a higher score indicates better performance to be in line with the rest of physical fitness components.

## Data Availability

Data are not available for sharing since we did not obtain children’s parents consent to widely share the data nor was it included in the IRB protocol.
