# Supplementary material for: Physical activity, sedentary time, and fitness in relation to brain shapes in children with overweight/obesity: Links to intelligence
Source: Scand J Med Sci Sports. Author manuscript; Available in PMC 2024 Jul 7. (PMC11227654; doi:10.1111/sms.14263)
Supplement: Supp Table 1 [file NIHMS1999349-supplement-Supp_Table_1.docx]

**Table S1.** Descriptive characteristics of the study sample.

|  |  | All (n=100) | Boys (n=60) |  | Girls (n=40) |  |  |
| --- | --- | --- | --- | --- | --- | --- | --- |
|  |  | *Mean ± SD* | *Mean ± SD* |  | *Mean ± SD* |  |  |
| Age (years) |  | 10.0 ± 1.1 | 10.2 ± 1.1 |  | 9.8 ± 1.1 |  |  |
| Weight (kg) |  | 55.8 ± 11.0 | 56.7 ± 10.7 |  | 54.5 ± 11.5 |  |  |
| Height (cm) |  | 143.9 ± 8.3 | 144.7 ± 7.4 |  | 142.8 ± 9.4 |  |  |
| Body mass index (kg/m^2^) |  | 26.7 ± 3.6 | 26.9 ± 3.8 |  | 26.5 ± 3.5 |  |  |
| Weight status (n, %)* |  |  |  |  |  |  |  |
| Overweight |  | 26, 26.0% | 16, 26.7% |  | 10, 25.0% |  |  |
| Obesity type 1 |  | 43, 43.0% | 27, 45.0% |  | 16, 40.0% |  |  |
| Obesity type 2 |  | 20, 20.0% | 10, 16.7% |  | 10, 25.0% |  |  |
| Obesity type 3 |  | 11, 11.0% | 7, 11.7% |  | 4, 10.0% |  |  |
| Peak height velocity (years) |  | 12.3 ± 0.7 | 12.8 ± 0.4 |  | 11.6 ± 0.3 |  |  |
| Parental university level (n, %) | | |  |  |  |  |  |
| Neither parent |  | 68, 66.0% | 43, 71.7% |  | 23, 57.5% |  |  |
| One parent |  | 18, 18.0% | 10, 16.7% |  | 8, 20.0% |  |  |
| Both parents |  | 16, 16.0% | 7, 11.7% |  | 9, 22.5% |  |  |
| **Physical activity (min/day)** **†** | |  |  |  |  |  |  |
| Light PA |  | 275.8 ± 39.5 | 270.6 ± 38.5 |  | 283.7 ± 40.2 |  |  |
| Moderate PA |  | 47.2 ± 17.4 | 53.1 ± 18.5 |  | 38.2 ± 10.5 |  |  |
| Vigorous PA |  | 7.8 ± 4.6 | 9.4 ± 4.8 |  | 5.2 ± 2.6 |  |  |
| MVPA |  | 54.9 ± 21.0 | 62.5 ± 22.3 |  | 43.4 ± 12.2 |  |  |
| **Sedentary time (min/day)** **†** |  | 561.1 ± 59.5 | 553.7 ± 59.8 |  | 572.3 ± 57.9 |  |  |
| **Physical fitness** | | |  |  |  |  |  |
| Cardiorespiratory fitness (VO_2_max) |  | 40.8 ± 2.8 | 40.8 ± 2.8 |  | 40.7 ± 2.8 |  |  |
| Upper-limb muscular strength (kg) |  | 16.6 ± 3.8 | 17.1 ± 3.9 |  | 15.9 ± 3.4 |  |  |
| Lower-limb muscular strength (cm) |  | 105.4 ± 18.6 | 106.6 ± 17.5 |  | 103.5 ± 20.2 |  |  |
| Speed-agility (sec) |  | 15.1 ± 1.6 | 14.9 ± 1.6 |  | 15.4 ± 1.5 |  |  |
| **Intelligence** |  |  |  |  |  |  |  |
| Crystallized intelligence |  | 103.2 ±12.7 | 102.8 ±12.3 |  | 103.7 ±13.5 |  |  |
| Fluid intelligence |  | 26.8 ± 5.0 | 26.4 ± 4.9 |  | 27.3 ± 5.0 |  |  |
| Total intelligence |  | 98.0 ± 11.9 | 96.7 ± 11.7 |  | 99.9 ± 12.0 |  |  |

SD= Standard deviation. VO_2_max= maximum oxygen volume. PA= physical activity. MVPA= moderate-to-vigorous physical activity.

Weight status and maternal education were presented as frequency and percentage. The rest of the sections were presented as mean ± standard deviation. For physical activity and sedentary time variables, sample size is n=98 (39 girls).

* Classified according to Cole et al.^1^ and Bervoets et al.^2^

† Classified according to Hildebrand et al.^3,4^ cut-off points for non-dominant wrist.

**Table S2.** Expansions in the right pallidum related to intelligence within those regions previously significant related to physical activity, sedentary time, and physical fitness.

|  | Contrast | Voxels |
| --- | --- | --- |
| **Fluid intelligence** |  |  |
| Upper-limb muscular strength | Expansions | 615 |
| Moderate physical activity | Expansions | 202 |
|  |  | 26 |
| Vigorous physical activity | Expansions | 441 |
|  |  | 4 |
| Moderate-to-vigorous physical activity | Expansions | 309 |
|  |  | 2 |
| **Total intelligence** |  |  |
| Upper-limb muscular strength | Expansions | 532 |
| Moderate physical activity | Expansions | 73 |
|  |  | 50 |
|  |  | 13 |
|  |  | 2 |
| Vigorous physical activity | Expansions | 304 |
|  |  | 17 |
| Moderate-to-vigorous physical activity | Expansions | 128 |
|  |  | 68 |
|  |  | 11 |

Expansions (i.e., positive associations) indicate larger radial distance (p < 0.05 threshold-free cluster enhancement corrected). Only regions that were previously significant correlated with physical activity, sedentary time, and physical fitness were included. All the analyses were adjusted for sex, peak height velocity, and parental education.

**REFERENCES**

1. Cole TJ, Lobstein T. Extended international (IOTF) body mass index cut-offs for thinness, overweight and obesity. *Pediatr Obes*. 2012;7(4):284-294. doi:10.1111/j.2047-6310.2012.00064.x

2. Bervoets L, Massa G. Defining morbid obesity in children based on BMI 40 at age 18 using the extended international (IOTF) cut-offs. *Pediatr Obes*. 2014;9(5):e94-e98. doi:10.1111/j.2047-6310.2014.00217.x

3. Hildebrand M, Hansen BH, Hees VT Van, et al. Evaluation of raw acceleration sedentary thresholds in children and adults. *Scand J Med Sci Sport*. 2017;27(12):1814-1823. doi:10.1111/sms.12795

4. Hildebrand M, VAN Hees VT, Hansen BH, Ekelund U. Age group comparability of raw accelerometer output from wrist- and hip-worn monitors. *Med Sci Sports Exerc*. 2014;46(9):1816-1824. doi:10.1249/MSS.0000000000000289
